# Diversity and Standard Nomenclature of Staphylococcus aureus Hyaluronate Lyases HysA and HysB

**DOI:** 10.1128/spectrum.00524-23

**Published:** 2023-06-26

**Authors:** Junzo Hisatsune, Yuma Koizumi, Kotaro Tanimoto, Motoyuki Sugai

**Affiliations:** a Antimicrobial Resistance Research Center, National Institute of Infectious Diseases, Tokyo, Japan; b Department of Antimicrobial Resistance, Hiroshima University Graduate School of Biomedical and Health Sciences, Hiroshima, Japan; c Department of Orthodontics and Craniofacial Developmental Biology, Hiroshima University Graduate School of Biomedical and Health Sciences, Hiroshima, Japan; University of Calgary

**Keywords:** *Staphylococcus aureus*, hyaluronate lyase, HysA, HysB, amino acid sequences

## Abstract

Bacterial hyaluronate lyases (Hys) are enzymes that degrade hyaluronic acid in their host and are known to contribute to the pathogenesis of several illnesses. The first two identified Hys genes in Staphylococcus aureus were registered as *hysA1* and *hysA2*. However, their annotations have been mistakenly reversed in some registered assembly data, and different abbreviations (*hysA* and *hysB*) in some reports complicates comparative analysis of Hys proteins. We investigated the *hys* loci of S. aureus genome sequences registered in public databases, analyzed the homology, and defined *hysA* as *hys* located in the core genome surrounded by a lactose metabolic operon and a ribosomal protein cluster present in almost all strains and *hysB* as that located on the genomic island νSaβ of the accessory genome. Homology analysis of the amino acid sequences of HysA and HysB revealed that they are conserved among clonal complex (CC) groups with a few exceptions. Thus, we propose a new nomenclature for S. aureus Hys subtypes: HysA_CC***_ for HysA and HysB_CC***_ for HysB, with the asterisks representing the clonal complex number of the S. aureus strain producing the Hys subtype. The application of this proposed nomenclature will facilitate the intuitive, straightforward, and unambiguous designation of Hys subtypes and contribute to enhancing comparative studies in this regard.

**IMPORTANCE** Numerous whole-genome sequence data for Staphylococcus aureus harboring two hyaluronate lyase (Hys) genes have been registered. However, the assigned gene names for *hysA1* and *hysA2* are incorrect in some assembled data, and in some cases, the genes are annotated differently as *hysA* and *hysB*. This creates confusion with respect to the nomenclature of Hys subtypes and complicates analysis involving Hys. In this study, we compared the homology of Hys subtypes and observed that to some extent, their amino acid sequences are conserved in each clonal complex group. Hys has been implicated as an important virulence factor, but relative sequence heterogeneity among S. aureus clones raises the question of whether Hys activities are different among these clones. Our proposed Hys nomenclature will facilitate comparison of the virulence of Hys, as well as discussions of the subject.

## OBSERVATION

Staphylococcus aureus, a commensal bacterial species that can colonize the skin and mucous membranes of the human body, can cause various infections, ranging from mild skin lesions to severe systemic infections (1).

Hyaluronate lyases (or hyaluronidases) are a group of enzymes that degrade hyaluronate, a high-molecular-weight, linear, unsulfated glycosaminoglycan polymer comprising alternating units of β-D-(1→3)-glucuronic acid and β-D-(1→3)-*N*-acetylglucosamine in the extracellular matrix. Furthermore, bacterial hyaluronate lyases (Hys), which act as endo-*N*-acetylhexosaminidases by eliminating the β-(1→4) linkage, resulting in the formation of a double bond in d-glucuronic acid (2), have been identified in various Gram-positive and -negative bacteria and have also been implicated in tissue penetration and invasion (2, 3). Jones et al. reported that proteomic analysis of the proteins secreted by S. aureus UAMS-1 led to the identification of two Hys proteins, encoded by *hysA1* and *hysA2* (4). Southern blot analysis of various strains further indicated that S. aureus possesses at least one *hysA* hybridizing band. In contrast, hybridizing bands have not been observed for other species of the genus Staphylococcus, suggesting that Hys is a potential determinant of the virulence of S. aureus (5). The strain UAMS-1 whole-genome sequence used in the study by Hart et al. (5) has been registered in a public database (6). The sequence similarity between the *hysA1* and *hysA2* genes of UAMS-1 (GenBank assembly accession no. GCA_000788115.1) was found to be 80.3%, while that between the HysA1 and HysA2 amino acid sequences of UAMS-1 was found to be 74.9%. Further, numerous whole-genome sequence data corresponding to S. aureus harboring two *hys* genes have been registered in the NCBI database (7). However, in some of the registered assembly data, the annotations of *hysA1* and *hysA2* were mistakenly reversed, and in some cases, they were annotated as *hysA* and *hysB*, respectively. This complicates the comparative analysis of Hys.

**FIG 1:**
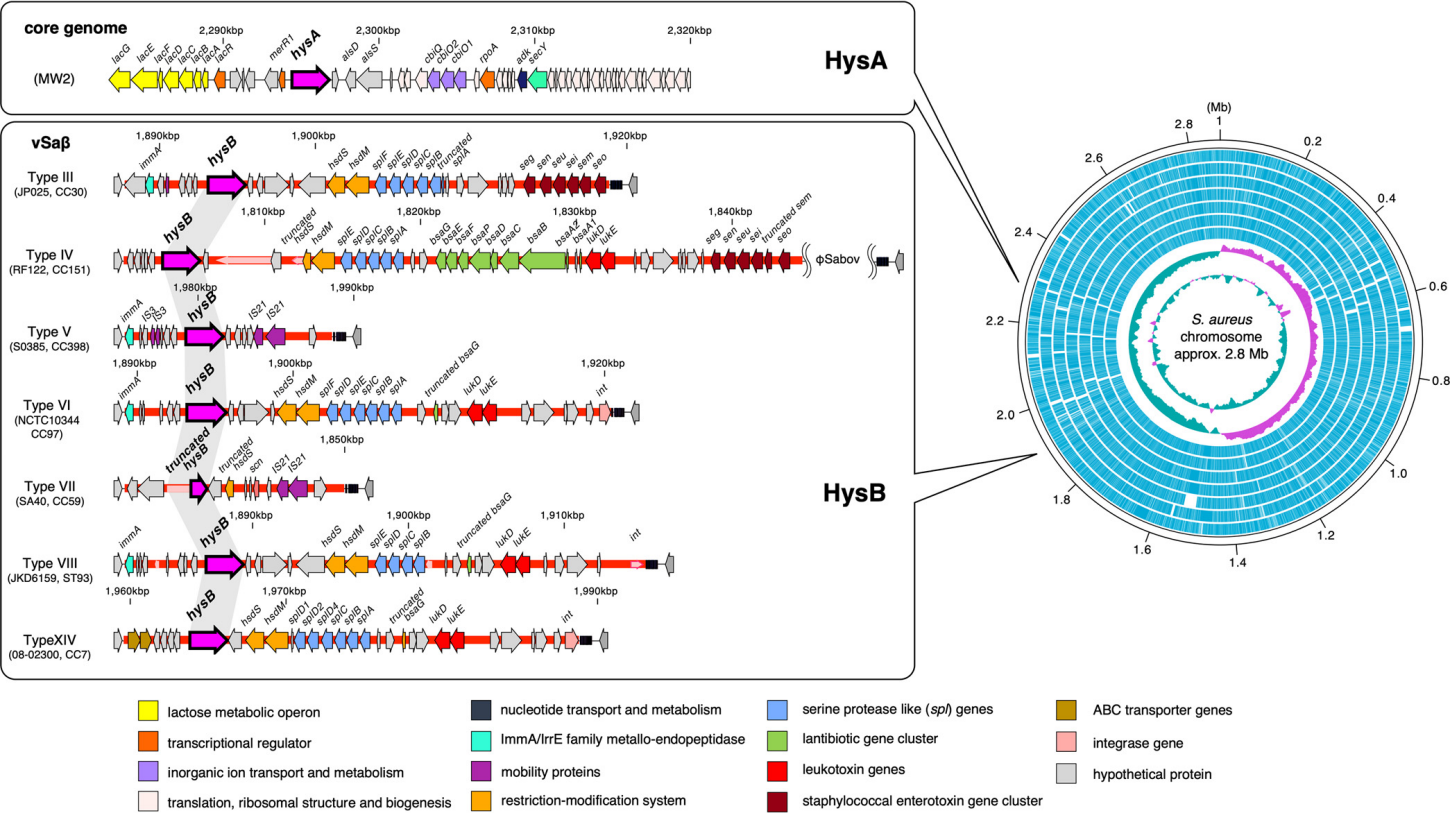
Gene map of S. aureus *hysA* and *hysB*. (Right) Circular map of seven S. aureus chromosomes harboring different *hysB*-positive νSaβ types. The complete genome sequence of S. aureus harboring *hysB*-positive νSaβ type XI was not registered in public databases, so it is not included in this figure. (Outer to inner) S. aureus JP025, RF122, S0385, NCTC10344, SA40, JKD6159, and 08-02300, respectively. (Left) Surrounding regions of *hysA* and *hysB*. The magenta arrows indicate *hysA* or *hysB*.

To clarify this issue, we investigated the *hys* loci of S. aureus genome sequences registered in public databases and analyzed the sequence similarity. As a result, we redefined the two *hys* genes (*hysA1* and *hysA2*) with low sequence similarity as “*hysA*” and “*hysB*.” The *hysA*- and *hysB*-flanking regions of representative strains are shown in Fig. 1. We defined *hysA* as *hys* located in the core genome surrounded by a lactose metabolic operon and the ribosomal protein cluster found in almost all strains. In contrast, we defined *hysB* as that located on the genomic island νSaβ of the accessory genome in a limited number of isolates. Klaui et al. classified νSaβ genomic islands into 15 different types and demonstrated their close correlation with clonal complexes (CCs) (8). Among them, eight νSaβ types (III, IV, V, VI, VII, VIII, XI, and XIV) were found to carry a sequence of *hysB in situ*, and of these, two νSaβ types (VII and XI) were found to possess a truncated form (Fig. 1).

**FIG 2:**
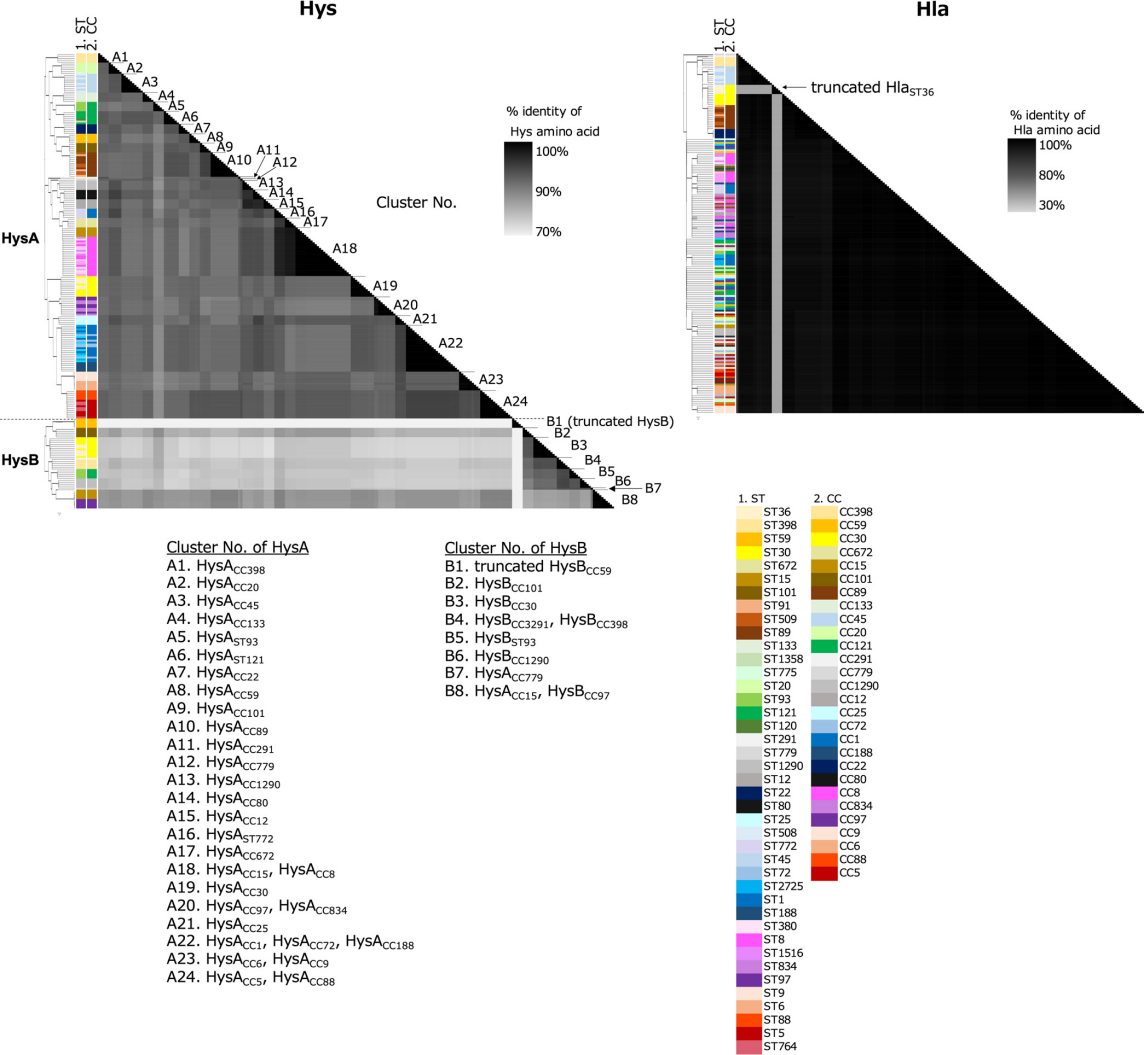
Pairwise average HysA, HysB, and Hla amino acid identity (AAI) heatmaps for various S. aureus sequence types (STs). In the HysA and Hla panels, the *x* and *y* axes indicate the 41 different STs of S. aureus. Identity scores (%) are displayed as black-gray gradations. The ST and CC types are depicted in columns to the left of the heatmaps.

We compared the amino acid sequences of HysA and HysB among the various CCs registered in public databases. The sequences used in this study are listed in Table S1 in the supplemental material. We aligned the HysA or HysB sequences of various sequence types (STs) using the ClustalW program with Molecular Evolutionary Genetic Analysis (MEGA) v10.1.5 (9). We then constructed a phylogenetic tree of the aligned amino acid sequences using RAxML-NG v1.0.1 (10), with the best model inferred using ModelTest-NG v0.1.7 (11) and 1,000 bootstrap replicates. Thus, comparative analysis revealed that S. aureus strains positive for HysA and those positive for HysB clustered separately, and each of the clusters shared high homology, forming a triangle (Fig. 2, left). The HysA amino acid sequences of CC8 and CC15 (cluster A18), and CC1, CC72, and CC188 (cluster A22) were 100% identical. CC97 and CC834 (cluster A20), CC6 and CC9 (cluster A23), and CC5 and CC88 (cluster A24) were over 98%, 98.7%, and 99.9% identical, respectively (Fig. 2, left). Therefore, we demonstrated that most *hysA* genes were conserved to some extent in each CC group. However, as an exception, the HysA sequence identity of ST772 belonging to CC1 and other STs in CC1 was 88.7%, and that between ST93 and ST121 belonging to CC121 was 87.3%. Thus, they were classified into individual clusters represented by the ST number. The HysB-positive CC groups were represented as CC15, CC30, CC59, ST93, CC97, CC101, CC779, CC398, and CC3291 (Fig. 2, left). Comparative analysis further revealed that the HysB amino acid sequence similarity between CC15 and CC97 (cluster B8) and between CC398 and CC3291 (cluster B4) was over 99.6% and 99.8%, respectively. In contrast, the HysB sequences of CC59 were truncated, exhibiting 36% sequence similarity among other CC groups (Table S1). Therefore, similarly to HysA, most of the HysB sequence was conserved among the CC groups.

We also compared the coding sequences of the α-hemolysin gene (*hla*), which is a ubiquitous virulence gene present in the genomic island, νSaγ, as a control (Fig. 2, right). Thus, we observed that the sequence similarity of Hla was highly conserved among various STs, showing over 98% identity in almost all the STs. The only exception was Hla in ST36, which exhibited a considerably lower similarity (35%), indicating that it was truncated in ST36.

Given that the absence of specificity or consistency in HysA or HysB nomenclature may lead to confusion in characterizing Hys-producing S. aureus, we propose a standard notation for a large variety of HysA and HysB. According to the nomenclature for staphylococcal superantigens (12), we recommend that *hys* genes be alphabetized in the order in which they are identified; therefore, a gene should be named *hysA* if it is located on the core genome and *hysB* if it is located on a mobile element. Further, we recommend naming new, protein-coding *hys* genes with 90% homology as “*hysA*_CC***_” for HysA subtypes and “*hysB*_CC***_” for HysB subtypes, with the asterisks indicating the clonal complex number of S. aureus. Hys has been implicated as one of the important virulence factors of S. aureus; however, the relative sequence heterogeneity among S. aureus clones raises the question of whether the observed Hys activities are different, as this may affect the virulence of S. aureus clones. Our study results and the proposed nomenclature will facilitate the intuitive, straightforward, and unambiguous designation of HysA or HysB genes and provide a platform for comparing the Hys activity in S. aureus clones in future studies. We may also need to consider the nomenclature of other virulence factors and enzymes of S. aureus with similar nomenclature conflicts in future studies.
